# The ****Suppressing Effects of Dkk3 Expression on Aggressiveness and Tumorigenesis of Colorectal Cancer

**DOI:** 10.3389/fonc.2020.600322

**Published:** 2020-11-30

**Authors:** Shuang Zhao, Chang-lai Hao, En-hong Zhao, Hua-mao Jiang, Hua-chuan Zheng

**Affiliations:** ^1^ Department of Oncology and Experimental Center, The Affiliated Hospital of Chengde Medical University, Chengde, China; ^2^ Department of Hematology, The Affiliated Hospital of Chengde Medical University, Chengde, China; ^3^ Department of Surgery, The Affiliated Hospital of Chengde Medical University, Chengde, China; ^4^ Department of Urology, The First Affiliated Hospital of Jinzhou Medical University, Jinzhou, China

**Keywords:** Dkk3, colorectal cancer, tumorigenesis, aggressive phenotypes, pathological behaviors, prognosis

## Abstract

Dkk3 has been discovered during comparison of immortalized and parental cells. Its expression has been shown to reduce colony formation and induce apoptosis of cancer cells, acting as a tumor suppressor. Herein, we demonstrate that Dkk3 overexpression or protein treatment may inhibit colorectal cancer cell proliferation, migration, and invasion and that they may promote apoptosis and G_2_ phase arrest with hypoexpression of Bcl-2, cdc25B, cdc25c, N-cadherin, slug, and twist and hyperexpression of Bax and E-cadherin. This effect is consistent with that of recombinant Dkk3 exposure and blocked with anti-Dkk3 antibody. Dkk3 deletion in intestinal cells was not associated with the emergence of epithelial lesions; however, adenoma emerged after sodium desoxycholate treatment. At both mRNA and protein levels, Dkk3 expression was higher in normal than in cancer tissues (*p*<0.05). *Dkk3* mRNA expression was negatively associated with its promoter methylation, growth pattern, differentiation, and favorable prognosis in the patients with colorectal cancer (*p*<0.05). *Dkk3*-related signal pathways in colorectal cancer included those of cellular adhesion and migration, melanogenesis, chemokine, Hedgehog, JAK-STAT, TOLL-like receptor, TGF-β, MAPK, and calcium signaling (*p*<0.05). These findings indicate that Dkk3 expression levels can help assess cancer aggressiveness and patient prognosis. It might also suppress aggressive phenotypes and tumorigenesis as a molecular target in gene therapy.

## Introduction

Dkk3 was discovered by representational difference PCR analysis; its mRNA expression has been shown as downregulated in immortalized cells compared to control (1). A homology alignment indicates that *Dkk3* is identical with reduced expression in immortalized cells (*REIC*). Dkk3 cDNA encodes a secreted glycoprotein with five potential N-glycosylation sites, an N-terminal signal peptide, two cysteine-rich domains, and two coiled-coil domains (2). TNF-α downregulated Dkk3 expression in normal skin keratinocytes and mouse skin and hair culture models, which was abrogated by anti-TNF-α antibody (3). In prostate cancer cells, Dkk3 expression inhibited cell proliferation and tumor growth, induced apoptosis, and sensitized cells to doxorubicin by c-JNK activation, mitochondrial redistribution of Bax, or by triggering Bcl-2 hypoexpression (4).

Previous studies have reported that Dkk3 induced JNK activation *via* endoplasmic reticulum (ER) stress and mitochondrial pathways (4–6). Takata et al. (7) demonstrated that DKK3 initiated apoptosis *via* mitochondrial and Fas death receptor pathways in mucinous ovarian carcinoma cells. Exogenous Dkk3 inhibited Wnt/β-catenin signaling and cell proliferation in kidney cancer cells (8), and the cysteine-rich core domain of Dkk3 was required for dendritic cell–like differentiation from monocytes and for tumor regression, where it activated phosphorylation of GSK-3β and stat (9). Meanwhile, adeno-Dkk3 virus has been shown to inhibit tumor growth and lymph node metastasis and prolong survival in nude mice with prostate cancer by inducing apoptosis, suppressing cell invasion and migration, and decreasing MMP-2 activity (10).

Dkk3 protein is expressed in the brain, heart, kidney, mammary gland, liver, pancreas, and lymph node (11). Its expression has been reported as downregulated in cancer cells, including in hepatocellular carcinoma, renal clear cell carcinoma, cervical squamous carcinoma, non-small cell lung cancer, and seminoma (12–17). In gastric cancer, Dkk3 overexpression or Dkk3 treatment decreased the karyoplasmic ratio, cell proliferation, migration, invasion, and lamellipodia formation and increased the likelihood of G_1_ phase arrest and apoptosis, which were blocked by anti-Dkk3 antibody. Dkk3 knockdown caused the opposite effect in immortalized gastric epithelial cells. Immunohistochemically, the level of Dkk3 expression was inversely correlated with tumor size, lymph node involvement, cell dedifferentiation, and unfavorable prognosis in gastric cancer. Moreover, serum Dkk3 concentration has been reported as remarkably higher in age-matched controls than in gastric cancer patients, where it was inversely associated with tumor size (18).

Colorectal cancer is a type of cancer associated with high risk of mortality (19, 20). Although colorectal adenoma pathologically and genetically precedes adenocarcinoma, its molecular mechanisms remain elusive. Previously, we have shown that Dkk3 expression was remarkably downregulated in colorectal non-neoplastic mucosa, adenoma to adenocarcinoma, and negatively correlated with invasion depth, TNM stage, and colorectal cancer cell dedifferentiation rate (21). Herein, we observed *in vitro* and *in vivo* the effects of Dkk3 expression and recombinant Dkk3 treatment on aggressive phenotypes of colorectal cancer cells, aiming to clarify the molecular mechanisms involved. Additionally, we established a mouse model of conditional *Dkk3* knockout in intestinal epithelial cells, using villin promoter to initiate cre recombination. Finally, pathological and bioinformatics analyses were performed to explore clinicopathological or prognostic significances of Dkk3 expression.

## Materials and Methods

### Cell Culture

Colorectal cancer cell lines (HCT-15 and HCT-116) were isolated from the same colorectal cancer patients with Duke’s C and purchased from the Cell Bank of Chinese Academy of Sciences, Shanghai, China. They were cultured in RPMI 1640 medium containing FBS, penicillin, and streptomycin in 5% CO_2_ at 37°C. Both kinds of cells were transfected with pCDNA3.1*-Dkk3* and pcDNA3.1 at 70% confluence 24 h after seeding on dishes according to the manufacturer’s instructions (QIAGEN, USA). Both HCT-15 and HCT-116 were treated with recombinant Dkk3 (R&D Systems, 1118-DK, USA) in RPMI 1640 at a dose of 50, 100, 150, or 200 ng/mL or their Dkk3 transfectants were exposed to anti-Dkk3 antibody (R&D Systems) at a dose of 30 or 60 ng/mL. Nontreated HCT-15 and HCT-116 cells were considered as control and pcDNA3.1 transfectant as mock.

### Immunofluorescence

Cells were attached to glass coverslips and fixed with 4% neutral formaldehyde and permeabilized with 0.2% Triton X-100. After washing with PBS, cells were incubated overnight at 4°C with goat anti-Dkk3 antibody (R&D system) and subsequently with antigoat IgG-FITC antibody (Santa Cruz) at room temperature and then stained with DAPI for nuclear labeling. Finally, we mounted coverslips with SlowFade^®^ Gold antifade reagent (Invitrogen) and observed them using a laser confocal microscope.

### ELISA Assay

Human Dkk-3 DuoSet ELISA Kit (R&D Systems) was employed to detect the Dkk3 level of cell culture supernatant and patient serum.

### Proliferation Assay

We used cell counting kit-8 (CCK-8) to determine cell proliferation. In brief, 2.0 × 10^3^ cells/well were cultured on a 96-well plate. After adhering to the plate, 10 μL of CCK-8 solution was added to each well at a different time point; absorbance was measured at 450 nm after 3 h incubation.

### Cell Cycle Analysis

We trypsinized, collected, and fixed the cell line using ethanol for 2 h. After RNase treatment for 1 h, cells were pelleted and stained by propidium iodide (PI) for 30 min. Finally, flow cytometry was used to examine the PI signal.

### Apoptosis Assay

FITC-labeled annexin V staining (BD Pharmingen) was employed to indicate phosphatidylserine externalization of early apoptosis. In brief, 1 × 10^6^ cells were collected, washed with PBS, and pelleted. FITC-labeled annexin V (5 μL, final concentration: 1 ug/mL) and PI (5 μL, final concentration: 50 µg/mL) were added to cell suspension, mixed, incubated for 15 min, and examined by flow cytometry.

### Mitochondiral Membrane Potential

We determined the mitochondrial membrane potential using JC-1 Mitochondrial Membrane Potential Assay Kit (Kagen, China). In brief, cells were stained by JC-1 (100 uL/mL) for 30 min and harvested for flow cytometry with JC-1 monomer (green, low concentration in cytosol) as FL1 channel and JC-1 aggregates (red, high concentration in mitochondria) as FL2 channel. In addition, the harvested cells were prepared as slides and observed under a fluorescence microscope.

### Transwell Chamber Assay

To assess the extent of cell invasion, we cultured 2.0 × 10^5^ cells in FBS-free medium in the matrigel-coated insert on the chamber top and added 10% FBS-containing medium to the chamber bottom as a chemoattractant. After 24 h incubation, the upper part of the insert was scrubbed, and the lower part was fixed in methanol, followed by Giemsa dye. To measure capacity for cell migration, we repeated this procedure without the membrane control insert.

### Animals

We housed three mice per plastic cage, which included paper chips, standard rodent food, and water provided in pathogen-free and temperature-controlled conditions with a 12-h light/dark illumination cycle. All experiments involving mice were approved by the Committee on Animal Experimentation of the Affiliated Hospital of Chengde Medical University. We performed cre- mediated deletion of floxed alleles in a germline by mating *Dkk3* conditional mutants (kindly presented by Prof. Kumon) with villin (intestine-specific)-cre mice (Jax Lab). At least 5 mice were sacrificed at 9 months, and their intestines were histologically analyzed. To chemically induce colorectal tumor, we orally administrated sodium desoxycholate in 0.09% agarose suspension to male villin-cre/*Dkk3* (*n*=5, 16–18 g, age 8 weeks) and wild-type (WT, *n*=5, 16–18 g, age 8 weeks) mice for 6 weeks. After 40 weeks, these mice were sacrificed, and their colorectal tissues were subjected to pathological examination, RNA, and protein extraction.

### Subjects

Colorectal cancer and paired normal mucosa (*n*=107) tissues were sampled from surgical resection specimens, acquired at the Affiliated Hospital of Chengde Medical University between 2017 and 2019. The patients with CRC were 59 men and 48 women (22~85 years, mean=62.9 years). Among them, 47 cases are accompanied with lymph node metastasis. These cases included well (*n*=19), moderately (*n*=60), and poorly (*n*=12) differentiated; mucinous (*n*=7); and other (*n*=9) adenocarcinomas. Serum samples were obtained before surgery from 13 patients with colorectal cancer and 40 healthy individuals attending Affiliated Hospital of Chengde Medical University. None of the patients had undergone chemotherapy, radiotherapy, or any other treatment before the operation. All participants provided comprehensive consent to participate in research and for their data to be used further. The study protocol was approved by the Ethics Committee of the Affiliated Hospital of Chengde Medical University.

### DNA Analysis

DNA was extracted from mouse tail and intestine using the phenol-chloroform method. We performed genotyping by PCR. The PCR primer sequences were CSD-lacF: 5’-GCTACCATTAC CAGTTGGTCTGGTGTC-3’, CSD-neoF: 5’-GGGATCTCATGCTGGAGTTCTTCG-3’, CSD- loxF:5’-GAGATGGCGCAACGCAATTAATG-3’, CSD*-Dkk3*-R:5’-AACAGGAGATTCCAGGT GTCAGAGG-3’, CSD*-Dkk3*-ttR: 5’-GCCTGGCCAGCACTTTTATCTATCC-3’, CSD*-Dkk3*-F: 5’-TCTGCTTTAGCCATACCTCTTGGGG-3’, *Dkk3*-F: 5’-ACCAAAGGTG GCAATGGGACC ATCT-3’, *Dkk3*-R: 5’-GGTGGAAAGCACTCTAAGGCCCAGC-3’, cre: 5’-GCCTGCATTACC GGTCGATGC-3’ and 5’-CAGGGTGTTATAAGCAATCCC-3’. The presence of truncated *Dkk3* and cre was confirmed by tail DNA PCR. *Dkk3* deletion was confirmed by PCR amplification of intestinal mucosa DNA.

### Real-Time RT-PCR

We extracted total RNA from cells and tissues using Trizol (Takara). Reverse transcription of 1 µg RNA was performed using random primers and AMV reverse transcriptase. PCR primers were designed according to the sequences provided by GenBank. The oligonucleotide primer forward sequence was 5’-ACAGCCACAGCCTGGTGTA-3’. The reversed primer sequences were 5’-CC TCCATGAAGCTGCCAAC-3’ for *Dkk3* (120 bp), 5’-TAGAATTGGTAGTTCTTCAT-3’ and 5’-A TTGCATCCCAGACAGTG-3’ for N-cadherin (100 bp), and 5’-CAGGTCTCCTCATGGCTT-3’ and 5’-CATCCTTAAATCTCACTCT -3’ for E-cadherin (140 bp). For internal control, *GAPDH*, forward primer sequences were 5’-CAATGACCCCTTCATTGACC-3’; reversed sequences were 5’-TGGAAGATGGTGATGGGATT-3’ (135 bp). SYBR Premix Ex Taq II kit (Takara) was used to amplify *GAPDH* as an internal control.

### Western Blot

Protein was extracted in RIPA lysis buffer and identified by BCA assay. We separated denatured protein in SDS-polyacrylamide gel and transferred it to the Hybond membrane, which was blocked with 5% milk in TBST. For immunoblotting, the membrane was incubated for 1 h with mouse anti-Bax (Santa Cruz), rabbit anti-cdc25B (H-85, Santa Cruz), anti-cdc25C (C-20, Santa Cruz), anti-E-cadherin (Cell Signaling Technology), anti-N-cadherin (Cell Signaling Technology), anti-slug (Cell Signaling Technology), anti-twist1 (Cell Signaling Technology), or anti-GAPDH (Santa Cruz) antibody for 1 h in TBST at room temperature. Subsequently, these membranes were incubated with antimouse or antirabbit IgG conjugated to horseradish peroxidase (Dako) for 1 h at room temperature. Bands were visualized using ECL- Plus (Santa Cruz).

### Histological Analysis

Tissues were subjected to routine pathological block preparation. Consecutive sections were deparaffinized, dehydrated, and subjected to immunostaining as previously described (18) or to the TUNEL procedure, using ApopTag Plus Peroxidase in Situ Apoptosis Detection Kit (Millipore) as recommended.

### Bioinformatics Analysis

The differences in level of Dkk3 expression were examined between colorectal normal and colorectal cancer tissues using Oncomine (www.oncomine.com). Data on the rate of expression (RNA-seqV2) and methylation as well as data on clinicopathological characteristics of colorectal cancer patients were extracted from the TCGA database using TCGA-assembler in R software. We integrated our raw data and examined Dkk3 expression levels in colorectal cancer tissue, which were subsequently examined against patient clinicopathological and prognostic characteristics. The Kaplan-Meier method was used to examine the prognostic significance of Dkk3 mRNA expression. Moreover, we used these data to examine the rate of promoter methylation and to perform GSEA. Based on Dkk3 expression levels, the data were divided into high- and low-expression groups based on the median value of colorectal cancer patients. GSEA was performed with GSEA-3.0. The impact of *Dkk3* methylation and expression on survival rates was examined using methylation, clinical, and transcriptome data. Methylated and unmethylated signals were employed to calculate β-coefficients, which are a quantitative representation of DNA methylation levels. Cutoff values were defined as median values of β-coefficients and mRNA expression levels. Kaplan-Meier survival curves of *Dkk3* methylation were obtained as well.

### Statistical Analyses

We repeated 3 different experiments to calculate mean and standard deviation. Statistical analysis was performed using Mann-Whitney U to differentiate the means and log-rank statistic to compare the survival rate. We used SPSS 10.0 for data analysis. *P*-values < 0.05 were considered indicative of statistical significance.

## Results

### Dkk3 Expression and Aggressive Colorectal Cancer Cell Phenotype

**Figure 1:**
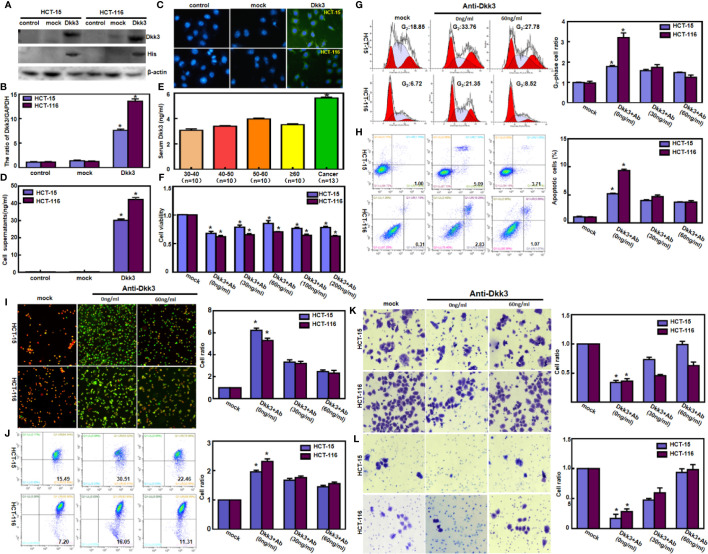
Dkk3 overexpression suppressed the aggressive phenotypes of colorectal cancer cells. After transfection of pcDNA3.1*-Dkk3*, Dkk3 expression became strong in HCT-15 and HCT-116 cells using anti-Dkk3 and anti-His antibodies by Western blot **(A)**, real-time RT-PCR **(B)**, and immunofluorescence (**C**, green: positive for Dkk3, blue: DAPI for nuclei). The cell culture supernatant of Dkk3 transfectants showed a higher Dkk3 concentration in comparison with those of the control and mock **(D)**. Colorectal cancer patients showed a higher serum Dkk3 level than healthy individuals regardless of their age, evidenced by ELISA assay **(E)**. The transfectants showed a lower proliferation **(F)** and G_2_ arrest **(G)** in comparison with the control and mock. There was an apoptosis-induced effect of Dkk3 overexpression in the transfectants of HCT-15 and HCT-116, evidenced by annexin V assay **(H)**. JC-1 staining displayed that the mitochondrial membrane potential was decreased in the abovementioned cells with Dkk3 overexpression by fluorescence **(I)** and flow cytometry **(J)**. Dkk3-overexpressing cells had a weaker ability to migrate **(K)** and invade **(L)**. We also treated the Dkk3 transfectants with anti-Dkk3 antibody (0, 30, and 60 ng/ml) and found that this antibody blocked the effects of Dkk3 overexpression on these phenotypes (**F–L**, *p* < 0.05). mock, cells transfected with pcDNA3.1 vector; *P < 0.05, compared with the control and treatment with anti-Dkk3 antibody.

After transfection with pcDNA3.1*-Dkk3*, HCT-15, and HCT-116 cells overexpressed Dkk3 protein by western blot using anti-Dkk3 or anti-His antibody (**Figure 1A**). In Dkk3 transfectants, mRNA levels increased 7- to 13-fold compared to those in the control cells (**Figure 1B**, *p*<0.05). Immunofluorescence testing showed higher levels of Dkk3 expression in the cytoplasm of transfectants than in that of control or mock cells (**Figure 1C**, *p*<0.05). The cell culture supernatant of Dkk3 tranfectants had a higher Dkk3 concentration than that of control or mock cells (**Figure 1D**, *p*<0.05). However, a higher level of Dkk3 expression was detected in the serum of colorectal cancer patients than in that of healthy controls regardless of age (**Figure 1E**, *p*<0.05). The rate of growth of Dkk3 transfectants was slower than that of control and mock cells (**Figure 1F**, *p*<0.05). Cell cycle analysis indicated G_2_ phase arrest in the transfectants but not in the control or mock cells (**Figure 1G**, *p*<0.05). There was a high rate of apoptosis in HCT-15 and HCT-116 cells after *Dkk3* transfection (**Figure 1H**, *p*<0.05),. JC-1 staining demonstrated that Dkk3 expression significantly reduced mitochondrial membrane potential in these cells (**Figures 1I, J**). Compared with control and mock cells, Dkk3 overexpression suppressed migration and invasion capacities of both colorectal cancer cell types (**Figures 1K, L**, *p*<0.05). Finally, treatment of Dkk3 transfectants with anti-Dkk3 antibody resulted in blocked effects of Dkk3 overexpression on these phenotypes (**Figures 1F–L**, *p*<0.05).

### Recombinant Dkk3 and Aggressive Colorectal Cancer Cell Phenotypes

**Figure 2:**
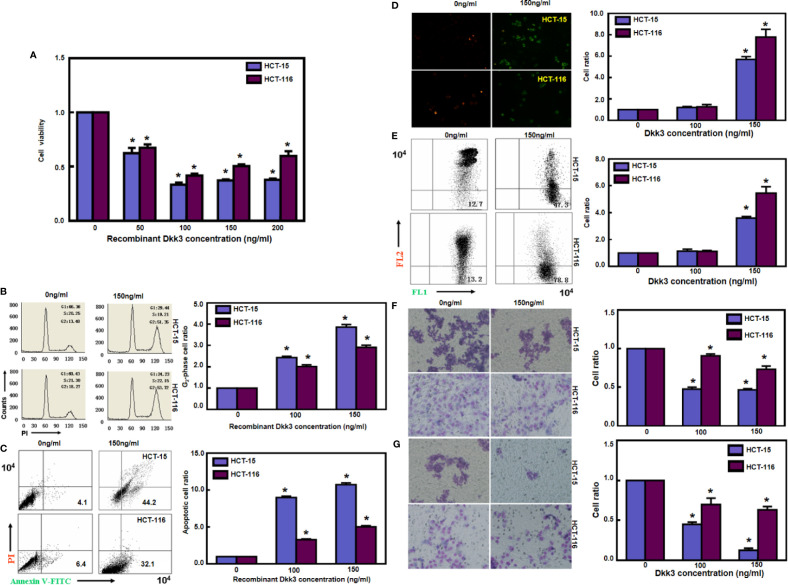
Recombinant Dkk3 inhibited the aggressive phenotypes of colorectal cancer cells. Treatment with recombinant Dkk3 protein significantly suppressed the proliferation of HCT-15 and HCT-116, compared with the control cells **(A)**. Recombinant Dkk3 protein could induce G_2_ arrest **(B)** and cell apoptosis **(C)** of both kinds of cells in a dose-dependent manner. JC-1 staining also demonstrated that recombinant Dkk3 exposure significantly reduced mitochondrial membrane potential of the abovementioned cells treated with 150 ng/mL recombinant protein by fluorescence **(D)** and flow cytometry **(E)**. After being exposed to recombinant Dkk3, both colorectal carcinoma cells exhibited a low ability to migrate **(F)** and invade **(G)**, compared with the control cells. *P < 0.05, compared with the control.

Treatment with recombinant Dkk3 protein significantly suppressed the proliferation of HCT-15 and HCT-116 cells (**Figure 2A**, *p*<0.05). Exposure to Dkk3 protein resulted in G_2_ phase arrest in HCT-15 and HCT-116 cells in a dose-dependent manner (**Figure 2B**, *p*<0.05). In addition, there was a higher level of apoptosis in HCT-15 and HCT-116 cells treated with Dkk3 protein (**Figure 2C**, *p*<0.05) than in the control cells. JC-1 staining also demonstrated that Dkk3 exposure significantly reduced the mitochondrial membrane potential of these cells (**Figures 2D, E**, *p*<0.05). In compare to control cells, Dkk3 protein exposure suppressed migration and invasion capacity of both colorectal cancer cell types (**Figures 2F, G**, *p*<0.05).

### Dkk3 and Expression of Phenotype-Related Proteins in Colorectal Cancer Cells

**Figure 3:**
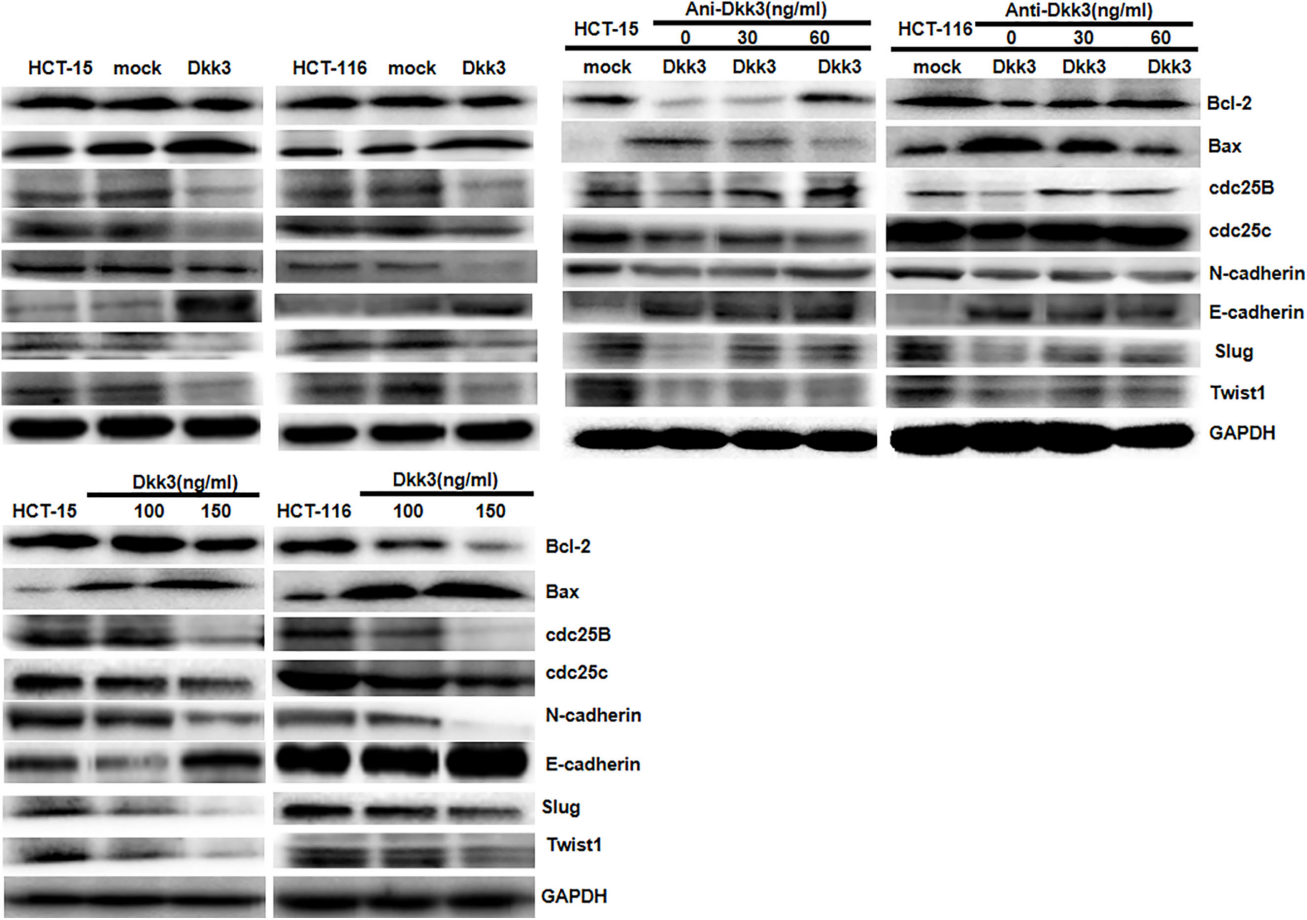
The phenotype-related proteins were screened by Western blot. Dkk3 overexpression and treatment decreased the expression of Bcl-2, cdc25B, cdc25c, N-cadherin, Slug, and Twist and increased the expression of Bax and E-cadherin in HCT-15 and HCT-116 cells. The anti-Dkk3 antibody suppressed the effects of Dkk3 overexpression on the expression of the phenotype-related proteins.

As indicated in **Figure 3**, Dkk3 overexpression and treatment decreased the levels of expression of Bcl-2, cdc25B, cdc25c, N-cadherin, slug, and twist; in contrast, it increased the levels of expression of Bax and E-cadherin in HCT-15 and HCT-116 cells. Use of anti-Dkk3 antibody suppressed the effects of Dkk3 overexpression on these phenotype-related proteins.

### The Effects of Dkk3 on Colorectal Carcinogenesis

**Figure 4:**
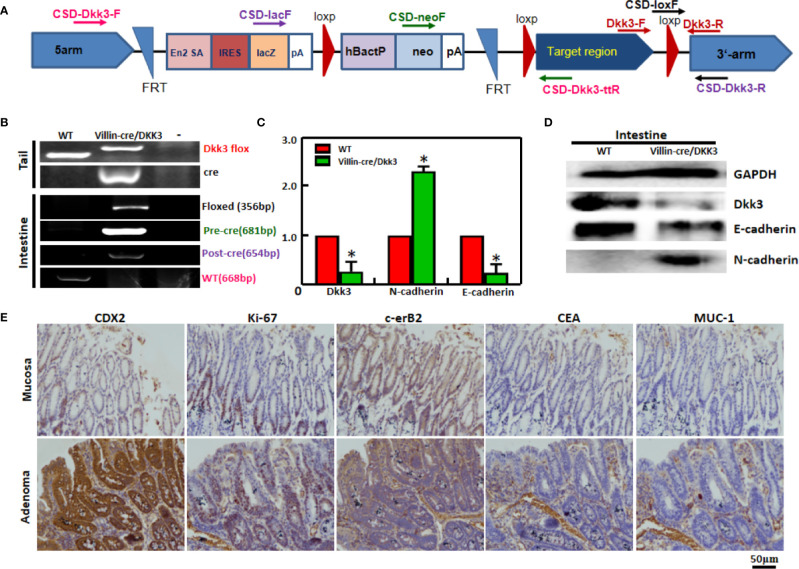
The effects of Dkk3 on tumor growth and carcinogenesis of colorectal cancer. PCR primers were designed **(A)** and subjected to PCR of tail and stomach DNA **(B)** with the same color for the corresponding primers and products. Expression of Dkk3, N-cadherin, and E-cadherin was confirmed by real-time PCR **(C)** and Western blot **(D)**. Immunohistochemistry was employed to observe the maker proteins’ expression in the intestinal adenoma of target villin-cre/Dkk3 knockout mice induced by sodium desoxycholate **(E)**. *p < 0.05, compared with the transfectant or knockout mice. WT, wild-type mice; VD, villin-cre +; Dkk3 -/-.

We matched the conditional *Dkk3*-KO mice with villin-cre mice and designed primers to confirm deletion of Dkk3 using DNA from tail and intestinal mucosa (**Figure 4A**). Using PCR, we confirmed biallelic deletion of *Dkk3* in intestinal mucosa of *Dkk3*-villin-cre mice (**Figure 4B**). Expression levels of Dkk3 and E-cadherin were downregulated in intestinal mucosa of Dkk3-villin-cre at both mRNA and protein levels compared to WT mice; the reverse was observed for N-cadherin expression (**Figures 4C, D**). However, no remarkable lesions were observed in the intestinal mucosa of either mouse group (data not shown). A single case of colorectal adenoma was observed in villin-cre/Dkk3 mice after chemical induction, showing a higher level of expression of CDX1, ki-67, and c-erB2 than that observed in normal mucosa (**Figure 4E**).

### Dkk3 Expression in Colorectal Cancer

**Figure 5:**
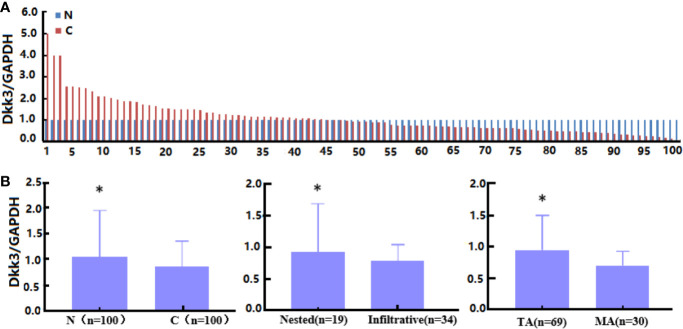
The clinicopathological significance of *Dkk3* mRNA expression in colorectal cancer. Real-time RT-PCR was employed to detect *Dkk3* mRNA expression in colorectal cancer samples **(A)**. Its expression was compared with pathological behaviors of colorectal cancer **(B)**. N, normal; C, cancer, TA, tubular adenocarcinoma; MA, mucinous adenocarcinoma. *p < 0.05.

*Dkk3* mRNA expression was examined in colorectal cancer tissues using RT-PCR (**Figure 5A**). *Dkk3* mRNA expression levels were lower in cancer than in normal tissues (**Figure 5B**, *p*<0.05). Concurrently, they were higher in tubular than in mucinous adenocarcinoma (**Figure 5B**, *p*<0.05). Levels of Dkk3 expression were higher in nested than in infiltrative adenocarcinomas (**Figure 5B**, *p*<0.05). However, these levels of expression were not correlated with gender, age, cell differentiation, lymph node or distal metastasis status, peritoneal spread, lymphatic and venous invasion, or tumor size in colorectal cancer patients (*p*>0.05, data not shown).

**Figure 6:**
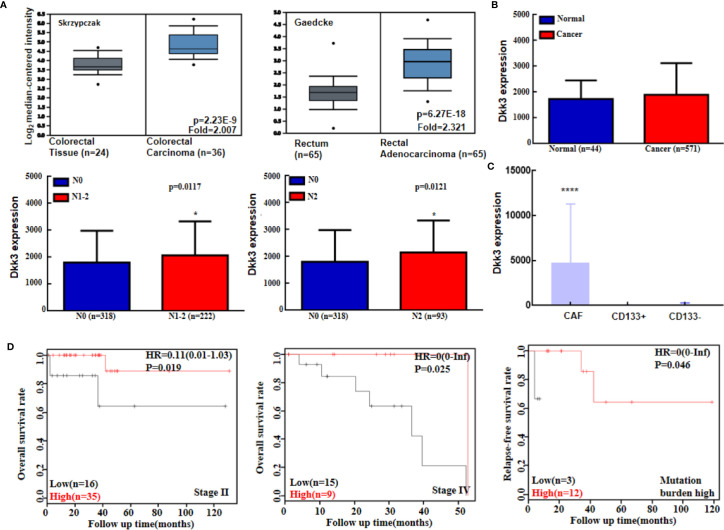
The bioinformatics analysis of *Dkk3* mRNA expression in colorectal cancer. Oncomine **(A)** and TCGA **(B)** data sets were employed to analyze Dkk3 expression in colorectal cancer, and its expression was compared with pathological parameters of cancers. *Dkk3* mRNA expression was higher in cancer-associated fibroblasts (CAF) than CD133-positive (CD133+) and -negative (CD133-) colorectal cancer cells **(C)**. Kaplan-Meier curves were used to analyze the prognostic significance of *Dkk3* mRNA expression according to KM plotters **(D)**. HR, hazard ratio; N, lymph node metastasis. *p < 0.05; ****p < 0.001.

Moreover, levels of *Dkk3* mRNA expression were higher in colorectal cancer than in normal mucosa according to the Skrzypczak and Gaedcke method (**Figure 6A**); however, no difference was detected in TCGA database analysis (**Figure 6B**). These levels were positively associated with lymph node metastasis status in colorectal cancer patients (**Figure 6B**). In addition, colorectal cancer-associated fibroblasts had a higher level of *Dkk3* expression compared to that observed in CD133+ or CD133- cancer cells (**Figure 6C**, *p*<0.05). There was no correlation between survival rate among colorectal cancer patients and *Dkk3* expression levels (*p*>0.05, data not shown). However, a negative relationship between *Dkk3* mRNA expression and prognosis was observed in patients with stage II or IV disease and in those with a high mutation burden (**Figure 6D**, *p<*0.05). Additionally, *Dkk3* mRNA expression levels were correlated negatively with the rate of promoter methylation (*p*<0.05, data not shown). There was no association between *Dkk3* promoter methylation and survival rates (*p*>0.05, data not shown).

**Figure 7:**
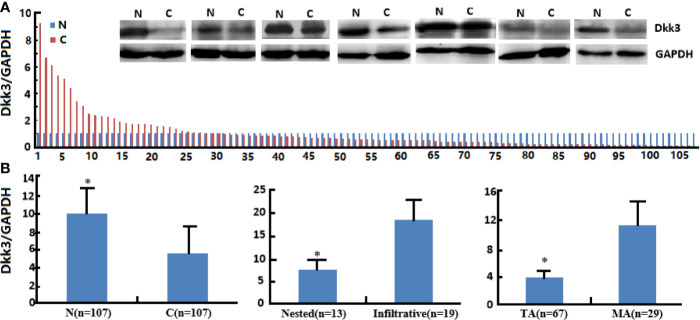
The clinicopathological significance of Dkk3 protein expression in colorectal cancer. Western blot was employed to detect Dkk3 protein expression in colorectal cancer samples **(A)**. Its expression was compared with pathological behaviors of colorectal cancer **(B)**. N, normal; C, cancer, TA, tubular adenocarcinoma; MA, mucinous adenocarcinoma. *p < 0.05.

Dkk3 protein expression levels were lower in cancer than in normal tissues (**Figure 7**, *p*<0.05). In addition, Dkk3 protein expression levels were lower in tubular than in mucinous adenocarcinomas (*p*<0.05); similarly, they were lower in nested than in infiltrative adenocarcinomas (*p*<0.05). However, Dkk3 protein expression levels did not correlate with gender, age, cell differentiation rate, lymph node or distal metastasis status, peritoneal spread, lymphatic and venous invasion, or tumor size in colorectal cancer patients (*p*>0.05).

### Dkk3-Related Signal Pathways in Colorectal Cancer

In GSEA, enriched *Dkk3*-related signaling pathways in colorectal cancer tissue included those associated with cellular adhesion and migration, melanogenesis, chemokine, Hedgehog, JAK-STAT, TOLL-like receptor, TGF-β, MAPK, and calcium signaling (**Table 1**, *p*<0.05).

**Table 1:** *Dkk3*-enriched signal pathway in colorectal cancer according KEGG analysis.

Pathway	Size	*P* value
Focal adhesion	197	<0.001
Gap junction	87	<0.001
Cytokine -cytokine receptor interaction	234	<0.001
Cell adhesion molecules CAMS	129	<0.001
Dilated cardiomyopathy	86	<0.001
Chemokine signaling pathway	183	<0.001
Hypertrophic cardiomyopathy	79	<0.001
Leukocyte transendothelial migration	111	<0.001
Hedgehog signaling pathway	54	<0.001
Regulation of actin cytoskeleton	207	<0.001
Melanogenesis	99	<0.001
ECM receptor interaction	83	<0.001
Axon guidance	127	<0.001
Pathways in cancer	322	<0.001
Basal cell carcinoma	54	<0.001
Arrhythmogenic right ventricular cardiomyopathy	71	0.001
Complement and coagulation cascades	64	0.001
Neuroactive ligand receptor interaction	231	0.001
JAK-STAT signaling pathway	130	0.001
Leishmania infection	70	0.005
TOLL-like receptor signaling pathway	87	0.006
Glycosaminoglycan biosynthesis chondroitin sulfate	22	0.006
Vascular smooth muscle contraction	113	0.006
Hematopoietic cell lineage	81	0.006
Calcium signaling pathway	169	0.007
TGF-β signaling pathway	85	0.009
MAPK signaling pathway	257	0.015
Glycosaminoglycan biosynthesis heparan sulfate	26	0.02
Melanoma	70	0.039

## Discussion

Shin et al. (22) reported that Dkk3 immunostaining was gradually downregulated from the epidermis in normal skin, actinic keratosis to squamous cell carcinoma (SCC). *Dkk3* mRNA expression level was lower in SCC than in normal skin. In the present study, expression of *Dkk3* mRNA and protein was downregulated in colorectal cancer cells, which is consistent with previous studies (12–17). A previous high-throughput study has shown a negative correlation between the level of *Dkk3* mRNA expression and its promoter methylation rate (18). These findings suggest that Dkk3 expression is downregulated in colorectal cancer due to Dkk3 promoter methylation. Our previous study of gastric and lung cancer cells treated with 5-Aza has shown a significantly negative association between Dkk3 promoter methylation and mRNA expression (18, 23). In a separate study, the rate of Dkk3 promoter methylation was higher in breast cancer than in normal tissue and was independently associated with adverse prognosis (24). Bioinformatics analysis has shown higher levels of *Dkk3* mRNA in colorectal cancer; however, this finding might be accounted by between-study differences in methods used (RT-PCR, Western blot, and tissue microarray vs. RNA sequencing). Herein, we have shown that colorectal adenoma occurred in chemical-exposed and Dkk3-deleted intestine, suggesting that Dkk3 abrogation might enhance chemically induced colorectal carcinogenesis.

Horikawa et al. (25) found that Dkk3 overexpression suppressed the proliferation of human bladder cancer cells by downregulating CD147 expression. Here, it was observed that both recombinant Dkk3 protein and Dkk3 overexpression might inhibit cell proliferation and trigger G_2_ phase arrest in both colorectal cancer cell types. Previously, we observed that the presence of Dkk3 overexpression and recombinant Dkk3 triggered G_1_ phase arrest in gastric cancer cells by downregulating Cyclin D2 and Cyclin E expression *via* the Wnt/β-catenin pathway (18). These data indicated that the effect of Dkk3 on the cell cycle depended on cell specificity. Additionally, a higher apoptosis rate was detected in both colorectal cancer cell types exposed to recombinant Dkk3 protein or transfected with Dkk3-expressing plasmid; this finding was consistent with that of our previous study (18). JC-1 staining showed that both treatments decreased mitochondrial membrane potential of colorectal cancer cells, suggesting both treatments induced colorectal cancer cell apoptosis *via* the mitochondrial pathway. Previously, we have reported on a positive relationship between Dkk3 and Caspase-3 expression in both colorectal and lung cancers (21, 23). It has been suggested that Dkk3 could cause c-JNK activation, mitochondrial translocation of Bax, and Bcl-2 under-expression in apoptotic induction (4). Shin et al. (22) have shown that Ad-Dkk3 induced JNK activation and subsequent apoptosis in lung cancer cells. It has been suggested that expression of cytosolic and secretory Dkk3 protein types might contribute to apoptotic induction of colorectal cancer cells *via* the mitochondrial pathway. Altogether, this evidence suggests that downregulated expression or loss of Dkk3 in colorectal cancer cells might disrupt the balance between apoptosis and proliferation.

In both gastric and colorectal cancers, Dkk3 expression was negatively correlated with the depth of invasion, lymph node involvement, and clinicopathological staging (18, 21); this finding was consistent with that of another study (26). Herein, the *Dkk3* mRNA expression level was higher in nested than in filtrated cancer tissue, and recombinant Dkk3 and ectopic Dkk3 expression seemed to attenuate the migrative and invasive capacities of colorectal cancer cells, indicating that both kinds of intervention might effectively inhibit the risk of invasion or metastasis in advanced colorectal cancer. Dkk3-related signal pathways included cellular adhesion and migration in colorectal cancer, supporting this hypothesis. Hoang et al. (27) have previously reported that Dkk3 can inhibit invasion and motility of osteosarcoma cells *via* the Wnt/β-catenin pathway. Meanwhile, adeno-Dkk3 gene delivery has been demonstrated to suppress tumor growth and the rate of invasion and metastasis of prostate cancer cells (10). Moreover, Dkk3 expression enhanced cellular adhesion and reduced the rate of cellular migration of melanoma cells (28). Than et al. (29) reported that intraperitoneal adeno-Dkk-3 administration suppressed peritoneal dissemination of scirrhous gastric cancer. These findings suggest that invasive and metastatic properties of malignancies might be due to decreased expression levels or loss of Dkk3.

Zenzmaier et al. (30) report that plasma Dkk3 level was increased among older adults and that it might serve as a marker of senescence. Concurrently, the serum level of Dkk-3 was significantly lower in ovarian, gastric, and colorectal cancer patients than in healthy controls (18, 31, 32). In contrast, serum Dkk3 concentration appeared higher in colorectal cancer patients than in healthy individuals; a similar finding was reported in a study of cervical cancer (32). In addition, we found higher supernatant Dkk3 level in Dkk3 transfectants of colorectal and gastric cancer cells (18). Dkk3 treatment caused the same results as Dkk3 overexpression in line with our data (18, 23), which could be blocked with anti-Dkk3 antibody (18). Zhang et al. (33) reported that soluble Dkk-3 protein levels were associated with prostate acinar growth. It has been proposed that soluble Dkk3 protein may bind to transmembrane co-receptor Lrp6 to suppress Wnt/β-catenin signaling (4, 34). As a result, we hypothesized that recombinant Dkk3 might bind to receptors to reverse aggressive phenotypes of gastrointestinal cancer cells in either an autocrine or a paracrine manner (17).

Dkk3 expression has been shown to induce β-catenin degradation by interacting with protoesome βTrCP, blocking the effect of nuclear β-catenin on TCF-4 activity, and suppressing the expression of TCF-4 targets (c-myc, VEGF and cylcin D) (17, 35). The human dynein light chain, Tctex-1, may interact with Dkk3, which might be involved in ER stress and intracellular dynein motor dynamics (36). However, a separate study has reported that Dkk3-carrying adenovirus infection of normal human fibroblasts induced interleukin-7 production, triggered by ER stress proteins (ASK1, p38, IRE1α and IRF-1) (37). The repressing effects of Dkk3 overexpression on aggressive colorectal cancer cell phenotypes might be due to a higher rate of secretion of its soluble form alongside interaction of its cytosolic and other types of protein.

In this study, hypoexpression of cdc25B and cdc25c was associated with the suppressing effects of Dkk3 on proliferation and tumor growth as cdc25B activates Cyclin-dependent kinase CDC2 required for mitosis progression; concurrently, cdc25C promotes Cyclin B-Cdk1 complex formation required for G_2_/M phase transition (38). The presence of Bax-Bcl-2 complex on the mitochondrial membrane can lower the rate of apoptosis as Bax can open the mitochondrial anion channel to initiate apoptosis (39). Consequently, Bax hyperexpression and Bcl-2 hyperexpression may explain the inductive effect of Dkk3 in apoptosis of colorectal cancer cells. Slug and twist promoted epithelial-mesenchymal transition (EMT) alongside E-cadherin under-expression and N-cadherin hyperexpression (40); downregulated expression of N-cadherin and of slug and twist, and upregulation of E-cadherin might result in the inhibitory effects of Dkk3 in EMT of colorectal cancer cells.

It has been reported that Dkk3 might orchestrate concomitant activation of β-catenin and YAP/TAZ in cancer-associated fibroblasts (CAFs) by interfering with the Kremen negative regulator and increasing cell-surface levels of LRP6 as an HSF1 effector, required to promote tumor aggressiveness (41). Zhou et al. (42) demonstrated that Dkk3 was produced by pancreatic stellate cells and stimulated tumor growth, metastasis, and chemoresistance of pancreatic ductal adenocarcinoma in the paracrine and autocrine manner *via* the NF-κB pathway. Zenzmaier et al. (43) found that Dkk-3 promoted fibroblast proliferation and myofibroblast differentiation and regulated angiopoietin-1 expression in prostatic stroma potentially by enhancing PI3K/Akt signaling, which triggered angiogenesis in prostate cancer. Our bioinformatics data showed higher *Dkk3* expression in CAF than in CD133+ or in CD133- cancer cells, supporting the hypothesis that Dkk3 in cancer stromal cells might be involved in cancer progression.

Dkk3 was positively associated with survival rate among patients with nasopharyngeal carcinoma (26). Yang et al. (23) found that *Dkk3* mRNA overexpression positively correlated with overall, progression-free, and post-progression survival rates among lung cancer patients even when stratified by sex, histological subtyping, disease grade, TNM stage, chemotherapy and radiotherapy type, or smoking status. Herein, Kaplan-Meier analysis indicated that mRNA expression or promoter methylation of *Dkk3* was not linked to overall survival rate among colorectal cancer patients; this finding was consistent with that of our immunostaining tests (21). Moreover, Dkk3 expression had no prognostic significance in colorectal cancer at either mRNA or protein level. However, its mRNA expression was negatively correlated with poor prognosis among colorectal cancer patients with Stage II and IV disease and among those with a high mutation burden; these findings might provide guidance for clinical practice.

In conclusion, Dkk3 expression was downregulated in colorectal cancer tissue due to its promoter methylation; this finding can be used in assessing cancer aggressiveness. Recombinant Dkk3 protein and forced Dkk3 overexpression might suppress aggressive phenotypes of colorectal cancer cells. These findings indicate that Dkk3 might be a promising target for gene therapy in colorectal cancer if it can specifically be expressed in cancer cells. However, future studies need to elucidate Dkk3 receptor or partner and the associated signaling pathway.

## Data Availability Statement

The raw data supporting the conclusions of this article will be made available by the authors, without undue reservation.

## Ethics Statement

The studies involving human participants were reviewed and approved by Affiliated Hospital of Chengde Medical University. The patients/participants provided their written informed consent to participate in this study. The animal study was reviewed and approved by Affiliated Hospital of Chengde Medical University. Written informed consent was obtained from the owners for the participation of their animals in this study.

## Author Contributions

SZ, C-lH, E-hZ, H-mJ, and H-cZ designed and carried out the experiment, and H-cZ wrote the manuscript. All authors contributed to the article and approved the submitted version.

## Funding

This study was supported by Award for Liaoning Distinguished Professor, and National Natural Scientific Foundation of China (81672700).

## Conflict of Interest

The authors declare that the research was conducted in the absence of any commercial or financial relationships that could be construed as a potential conflict of interest.****
